# A Bivalent Securinine Compound SN3-L6 Induces Neuronal Differentiation via Translational Upregulation of Neurogenic Transcription Factors

**DOI:** 10.3389/fphar.2018.00290

**Published:** 2018-04-05

**Authors:** Yumei Liao, Xiaoji Zhuang, Xiaojie Huang, Yinghui Peng, Xuanyue Ma, Zhi-Xing Huang, Feng Liu, Junyu Xu, Ying Wang, Wei-Min Chen, Wen-Cai Ye, Lei Shi

**Affiliations:** ^1^JNU-HKUST Joint Laboratory for Neuroscience and Innovative Drug Research, Jinan University, Guangzhou, China; ^2^College of Pharmacy, Jinan University, Guangzhou, China; ^3^Key Laboratory of Gene Engineering of the Ministry of Education, State Key Laboratory of Biocontrol, School of Life Sciences, Sun Yat-sen University, Guangzhou, China; ^4^Department of Neurobiology, Key Laboratory of Medical Neurobiology of Ministry of Health of China, Zhejiang Province Key Laboratory of Neurobiology, Zhejiang University School of Medicine, Hangzhou, China

**Keywords:** neural progenitor cell, protein synthesis, neurite outgrowth, neurogenesis, securinine, stem cell, transcription factor, translation

## Abstract

Developing therapeutic approaches that target neuronal differentiation will be greatly beneficial for the regeneration of neurons and synaptic networks in neurological diseases. Protein synthesis (mRNA translation) has recently been shown to regulate neurogenesis of neural stem/progenitor cells (NSPCs). However, it has remained unknown whether engineering translational machinery is a valid approach for manipulating neuronal differentiation. The present study identifies that a bivalent securinine compound SN3-L6, previously designed and synthesized by our group, induces potent neuronal differentiation through a novel translation-dependent mechanism. An isobaric tag for relative and absolute quantitation (iTRAQ)-based proteomic analysis in Neuro-2a progenitor cells revealed that SN3-L6 upregulated a group of neurogenic transcription regulators, and also upregulated proteins involved in RNA processing, translation, and protein metabolism. Notably, puromycylation and metabolic labeling of newly synthesized proteins demonstrated that SN3-L6 induced rapid and robust activation of general mRNA translation. Importantly, mRNAs of the proneural transcription factors Foxp1, Foxp4, Hsf1, and Erf were among the targets that were translationally upregulated by SN3-L6. Either inhibition of translation or knockdown of these transcription factors blocked SN3-L6 activity. We finally confirmed that protein synthesis of a same set of transcription factors was upregulated in primary cortical NPCs. These findings together identify a new compound for translational activation and neuronal differentiation, and provide compelling evidence that reprogramming transcriptional regulation network at translational levels is a promising strategy for engineering NSPCs.

## Introduction

Neuronal differentiation, including neurogenesis and the following neurite outgrowth, is a highly dynamic process that requires biogenesis of a series of critical proteins to achieve the transitions through different cellular states. Promoting neuronal differentiation is an important step in stem cell therapy and neural regeneration, which facilitates the reconstruction of neural circuits after neurodegeneration and brain injury. Through genetic manipulation of a specific subset of transcription factors, or chemical approaches that using single or a cocktail of small-molecule compounds to regulate gene expression of transcription factors, scientists have successfully reprogrammed NSPCs, induced pluripotent stem cells (iPSCs) or glial cells into different neuronal subtypes (Pang et al., 2011; Panman et al., 2011; Chambers et al., 2012; Guo et al., 2014; Heinrich et al., 2014; Zhang et al., 2015).

Increasing evidence has suggested that translational regulation, including mRNA localization, ribosome biogenesis and the translation initiation or elongation control, is another critical way to remodel the transcription factor network for neuronal differentiation (Kusek et al., 2012; Vessey et al., 2012; Hartman et al., 2013; Kraushar et al., 2013, 2014; Roffe et al., 2013; MacNicol et al., 2015; Werner et al., 2015; Popovitchenko et al., 2016). These recent findings lead to a possible alternative strategy for inducing or manipulating neuronal differentiation, which is to target translation-dependent regulation of transcription factors or other cell fate determinants. Indeed, a reversible photoregulation of translation of H-Ras mRNA has already proven as an effective approach to control neurite outgrowth (Ogasawara, 2014). However, despite the potential advantage that translation probably leads to quicker and more efficient control of protein levels without modifications of the host genome, it has not been reported thus far whether chemical induction of translation by small molecules is possible to promote neuronal differentiation.

**FIGURE 1:**
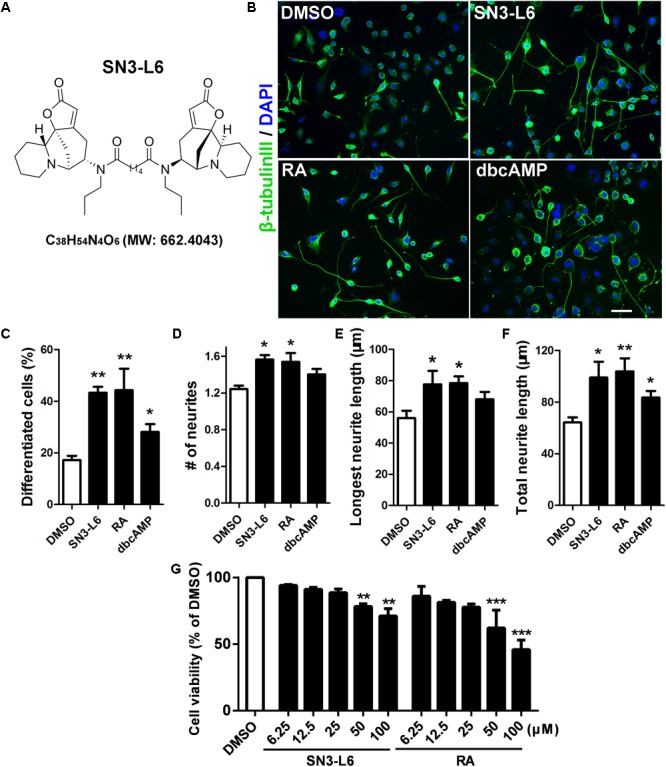
SN3-L6 potently promotes differentiation and neurite outgrowth. **(A)** The chemical structure, the chemical formula and the exact mass of SN3-L6 are shown. **(B)** Representative images of Neuro-2a cells after treatment with SN3-L6 (25 μM), RA (10 μM), or dibutyl cyclic AMP (dbcAMP) (0.5 mM) for 48 h. Scale bar, 50 μm. Neurites and nuclei were visualized using β-tubulin III antibody (green) and 49,6-diamidino-2-phenylindole (DAPI) (blue), respectively. Differentiation rate (% of cells that possess at least one process longer than 40 μm; **C**), neurite number **(D)**, longest neurite length **(E)**, and total neurite length **(F)** were quantified. ^∗^*P* < 0.05, ^∗∗^*P* < 0.01, indicated compound vs. DMSO. **(G)** Neuro-2a cells were treated with SN3-L6 or RA in a concentration gradient (6.25, 12.5, 25, 50, and 100 μM) for 48 h. Cell viability was tested using MTT assay. ^∗∗^*P* < 0.01, ^∗∗∗^*P* < 0.001, SN3-L6 or retinoic acid (RA) vs. DMSO. All data shown in this figure are presented as mean ± SEM from at least three independent experiments. Statistical analysis was subjected to one-way ANOVA with Bonferroni multiple comparison test.

We previously identified a securinine-derived compound, SN3-L6 (previously named compound **14**), which exhibits potent activities toward neurite formation of neuronal cells (Tang et al., 2016). Securinine is a natural compound that has long been suggested to have neuroexcitatory activities (Beutler et al., 1985). SN3-L6 possesses two moieties of securinine which linked by an *N^1^,N*^6^-dipropyladipamide (**Figure 1A**). Preliminary mechanistic studies showed that treatment of SN3-L6 quickly leads to activation of a common set of signaling pathways involved in neuronal differentiation, such as those dependent on mitogen-activated protein kinase (MAPK) or protein kinase B (Akt) (Tang et al., 2016). This suggests that SN3-L6 is capable of inducing rapid cellular changes for neuronal differentiation. However, on what molecular targets SN3-L6 acts and through what mechanism it governs cell fate and morphological changes have remained to be explored.

To further elucidate the molecular actions of SN3-L6 and its impact on cellular remodeling during neuronal differentiation, we performed an iTRAQ-based proteomic analysis to seek for unraveling a global protein change profile during early stages of SN3-L6 treatment. The result revealed that proteins in transcription regulation, mRNA translation and protein metabolism were upregulated. Notably, SN3-L6 led to a robust induction of global mRNA translation, and four proneural transcription factors Foxp1, Foxp4, Hsf1, and Erf were among the targets that are translationally upregulated. Finally, we confirmed that SN3-L6-stimulated neural induction is dependent on translation and synthesis of the transcription factors. Together, this study identifies a new translation-activating compound SN3-L6, which promotes neuronal differentiation through translational upregulation of a group of proneural transcription factors.

## Materials and Methods

### Antibodies and Reagents

SN3-L6 was synthesized as previously described and the purity was confirmed to be more than 98% by high-performance liquid chromatography (HPLC) (Tang et al., 2016). RA and dbcAMP were purchased from Sigma-Aldrich (St. Louis, MO, United States); Puromycin was from Life Technologies; Minimum Eagle’s medium (MEM), DMEM/F12, fetal bovine serum (FBS), trypsin, penicillin-streptomycin, B-27 supplement, N2 supplement, bFGF and EGF were from Gibco; DNase I was from Roche Diagnostics. All solvents used were of HPLC grade. The water used in this study was provided by a Milli-Q water purification system from Millipore (Billerica, MA, United States).

The following primary antibodies and inhibitors were used: antibodies against β-tubulin III and α-tubulin were purchased from Sigma-Aldrich; Antibodies against puromycin and MAP2 were from Millipore; Rabbit monoclonal antibodies against Foxp1, Hsf1, β-actin, total or phosphorylated forms of mTOR, S6K, S6, 4EBP1, eEF2K, eEF2, and eIF2α were from Cell Signaling Technology (Beverly, MA, United States); Foxp4, Erf, and GAPDH antibodies were from Abcam (Cambridge, United Kingdom). Pharmacological inhibitors LY294002, 4EGI1, and KN93 were purchased from Millipore; Rapamycin, cycloheximide, and anisomycin from Sigma-Aldrich; U0126 was from Cell Signaling Technology.

### Cell Culture and Protein Harvest

Neuro-2a cells (ATCC, Manassas, VA, United States) were cultured in MEM supplemented with 10% FBS as described previously (Xiao et al., 2013). For induction of neuronal differentiation, the medium was changed to MEM supplied with 0.5% FBS in the presence of SN3-L6 (25 μM), RA (10 μM), or dbcAMP (0.5 mM) for 48 h.

Primary cortical NPCs were cultured from embryonic day (E) 13.5 ICR mouse as previously described (Xiang et al., 2017). The use of animals was approved by the Ethics Committee on Animal Experiments at Jinan University, China, and was strictly performed according to the guidelines of the Care and Use of Laboratory Animals. All efforts were made to minimize the suffering and the number of animals used. Briefly, the cortices of E13.5 ICR mouse embryos were isolated, minced and incubated in a solution which containing 0.05% trypsin and 0.15% DNase I at 37°C for 15 min and then triturated to obtain individual cell suspension. Then, cells were cultured in DMEM/F12 supplemented with 2% B-27, 1% N2, 20 ng/mL EGF and 20 ng/mL bFGF to grow into neurospheres. For SN3-L6 induced neuronal differentiation assay, neurospheres were separated into single-cell suspension and seeded on matrigel (Becton Dickinson)-coated coverslips at a density of 1 × 10^4^ cells per 12 mm coverslip. After 24 h seeding, the culture medium was switched into DMEM/F12 with 2% B-27 supplement, 1% N2 supplement, 20 ng/mL cAMP and 20 ng/mL BDNF in the present of SN3-L6 for additional 5 days. For Western blot analysis, single-cell suspension was seeded in matrigel-coated 35-mm dishes at a density of 6 × 10^5^ cells/dish.

### Protein Digestion and iTRAQ Labeling

Neuro-2a cells were seeded in 100 mm dishes at a density of 6 × 10^5^ cells/dish. After 24 h culturing, DMSO or SN3-L6 was added to the cells for 2 h, and the cells were lysed for protein harvest. Protein digestion was performed according to the FASP procedure described by Wisniewski et al. (2009) and the resulting peptide mixture was labeled using the 8-plex iTRAQ reagent according to the manufacturer’s instructions (Applied Biosystems). Briefly, 200 μg of proteins of each sample were incorporated into 30 μL STD buffer (4% SDS, 100 mM DTT, 150 mM Tris–HCl pH 8.0). The detergent, DTT and other low-molecular-weight components were removed using UA buffer (8 M Urea, 150 mM Tris–HCl pH 8.0) by repeated ultrafiltration (Microcon units, 30 kD). Then 100 μL 0.05 M iodoacetamide in UA buffer was added to block reduced cysteine residues and the samples were incubated for 20 min in darkness. The filters were washed with 100 μL UA buffer three times and then 100 μL DS buffer (50 mM triethylammonium bicarbonate at pH 8.5) twice. Finally, the protein suspensions were digested with 2 μg trypsin (Promega) in 40 μL DS buffer overnight at 37°C, and the resulting peptides were collected as a filtrate. The peptide content was estimated by UV light spectral density at 280 nm using an extinctions coefficient of 1.1 of 0.1% (g/l) solution that was calculated on the basis of the frequency of tryptophan and tyrosine in vertebrate proteins.

For labeling, each iTRAQ reagent was dissolved in 70 μL of ethanol and added to the respective peptide mixture. The samples were labeled as (Sample1)-114, (Sample2)-115, (Sample3)-116, and (Sample4)-117, (Sample5)-118, (Sample6)-119, and were multiplexed and vacuum dried.

### Peptide Fractionation With Strong Cation Exchange (SCX) Chromatography

Isobaric tag for relative and absolute quantitation labeled peptides were fractionated by SCX chromatography using the AKTA Purifier system (GE Healthcare). The dried peptide mixture was reconstituted and acidified with 2 mL buffer A (10 mM KH_2_PO_4_ in 25% of ACN, pH 2.7) and loaded onto a PolySULFOETHYL 4.6 mm × 100 mm column (5 μm, 200 Å, PolyLC Inc., Columbia, MD, United States). The peptides were eluted at a flow rate of 1 mL/min with a gradient of 0–10% buffer B (500 mM KCl, 10 mM KH_2_PO_4_ in 25% of ACN, pH 2.7) for 2 min, 10–20% buffer B for 25 min, 20–45% buffer B for 5 min, and 50–100% buffer B for 5 min. The elution was monitored by absorbance at 214 nm, and fractions were collected every 1 min. The collected fractions (about 30 fractions) were finally combined into 10 pools and desalted on C18 Cartridges (Empore^TM^ SPE Cartridges C18 (standard density), bed I.D. 7 mm, volume 3 mL, Sigma). Each fraction was concentrated by vacuum centrifugation and reconstituted in 40 μl of 0.1% (v/v) trifluoroacetic acid. All samples were stored at -80°C until LC-MS/MS analysis.

### Liquid Chromatography (LC) – Electrospray Ionization (ESI) Tandem MS (MS/MS) Analysis by Q Exactive

Experiments were performed on a Q Exactive mass spectrometer that was coupled to Easy nLC (Proxeon Biosystems, now Thermo Fisher Scientific). 10 μL of each fraction was injected for nanoLC-MS/MS analysis. The peptide mixture (5 μg) was loaded onto a the C18-reversed phase column (Thermo Scientific Easy Column, 10 cm long, 75 μm inner diameter, 3 μm resin) in buffer A (0.1% Formic acid) and separated with a linear gradient of buffer B (80% acetonitrile and 0.1% Formic acid) at a flow rate of 250 nL/min controlled by IntelliFlow technology over 140 min. MS data was acquired using a data-dependent top10 method dynamically choosing the most abundant precursor ions from the survey scan (300–1800 m/z) for HCD fragmentation. Determination of the target value is based on predictive Automatic Gain Control (pAGC). Dynamic exclusion duration was 60 s. Survey scans were acquired at a resolution of 70,000 at m/z 200 and resolution for HCD spectra was set to 17,500 at m/z 200. Normalized collision energy was 30 eV and the under fill ratio, which specifies the minimum percentage of the target value likely to be reached at maximum fill time, was defined as 0.1%. The instrument was run with peptide recognition mode enabled.

### Sequence Database Searching and Data Analysis

MS/MS spectra were searched using MASCOT engine (Matrix Science, London, United Kingdom; version 2.2) embedded into Proteome Discoverer 1.3 (Thermo Electron, San Jose, CA, United States) against UniProt Mouse database (133549 sequences, download at March 3, 2013) and the decoy database. For protein identification, the following options were used. Peptide mass tolerance = 20 ppm, MS/MS tolerance = 0.1 Da, Enzyme = Trypsin, Missed cleavage = 2, Fixed modification: Carbamidomethyl (C), iTRAQ4/8plex (K), iTRAQ4/8plex (N-term), Variable modification: Oxidation (M), FDR ≤ 0.01. The mass spectrometry proteomics data have been deposited to the ProteomeXchange Consortium via the PRIDE partner repository with the dataset identifier PXD009180. Proteomic analysis of the up- and down-regulated proteins was performed using PANTHER Classification System^1^ and DAVID Bioinformatics Resources 6.8^2^.

### Polysome Profiling

Neuro-2a cells seeded in 100 mm dishes were grown to ∼80% confluence and treated with SN3-L6 (25 μM) or DMSO for 2 h. Cells were washed twice with ice-cold PBS plus 100 μg/mL cycloheximide. Cells were then collected in polysome lysis buffer, which is constituted of 0.3 mM NaCl, 15 mM MgCl_2_⋅6H_2_O, 15 mM Tris–HCl (pH 7.4), 20 mM DTT, 140 U/mL RNase inhibitor (Rnasin, Promega), 100 μg/mL cycloheximide and 1% Triton^®^ X-100. The samples were kept on ice for at least 10 min and then centrifuged at 17000 × *g* 5 min at 4°C. Total RNA levels were determined by using a NanoDrop spectrophotometer (Thermo Scientific).

The 10–50% sucrose density gradients made in 15 mM MgCl_2_⋅6H_2_O, 15 mM Tris–HCl (pH 7.4) and 0.3 mM NaCl were prepared fresh using a gradient former (Gradient Master, BioComp Instruments, Canada). The supernatant was layered onto the sucrose gradient and was centrifuged in an SW-41Ti rotor (Beckman Coulter) at 39,000 rpm at 4°C for 1.5 h. Gradient solutions from top to bottom were passing through a Piston Gradient Fractionator (BioComp Instruments, Canada) while RNA was detected at UV absorbance 260 nm. Then fractions were collected for RT-qPCR analysis.

### RT-qPCR

Total RNA or RNA from the polysome fractions was isolated using RNAiso Plus (TaKaRa) following the manufacturer’s suggested procedure. RT-qPCR was performed as we previously reported (Xiang et al., 2016). In brief, reverse transcription was performed to obtain cDNA products using M-MLV reverse transcriptase (Promega) and oligo(dT) primers. Then, the samples were mixed with iQ SYBR^®^ Green (Bio-Rad) and qPCR was performed using Roche LightCycler 480. Primers used in this study were as follows:

*foxp1* forward, 5′-GCTTCAACTTCTCCAACAGCAA-3′,
*foxp1* reverse, 5′-GCAATCATGCCTTGAGCGA-3′;
*foxp4* forward, 5′-CATCTCCTCAGAGCTTGCCC-3′,
*foxp4* reverse, 5′-GGCGGATAAGGGAAGCGTAG-3′;
*hsf1* forward, 5′-CATGCCCAGCAGCAAAAAGT-3′,
*hsf1* reverse, 5′-GTCGACCATACTTGGGCAC-3′;
*erf* forward, 5′-GACTACGGGGAGTTTGTCATC-3′,
*erf* reverse, 5′-AACCGTTTCCCCTTGGTC-3′;
*gapdh* forward, 5′- GAAGGTCGGTGTGAACGGAT-3′,
*gapdh* reverse, 5′-TTCCCATTCTCGGCCTTGAC-3′;
*etf1* forward, 5′-GTGGTAGCGGTGGGAAAACT-3′,
*etf1* reverse, 5′-AAACCCAATGCACCCTACCA-3′.

### Protein Synthesis Measuring Assays

Three methods were used to monitor the rate of mRNA translation. First, puromycin labeling of nascent polypeptides was detected by immunoblotting (Schmidt et al., 2009). Briefly, at the last 30 min of SN3-L6 treatment, puromycin (1 μM) was added to cells for pulse labeling. Cells were washed once with cold PBS and then lysed with RIPA buffer for protein harvest. The protein samples were then subjected to Western blot analysis using puromycin antibody.

Alternatively, OPP was used to image protein synthesis using Click-iT^®^ Plus OPP Protein Synthesis Assay Kit (Life Technologies). Briefly, cells were treated with SN3-L6 for 2 h. During the last 30 min of treatment, OPP (20 μM) was added to cells to label nascent peptides. Cells were washed once with PBS, and were then fixed in 3.7% formaldehyde for 15 min at room temperature. Cells were then permeabilized with 0.5% Triton^®^X-100 in PBS for 15 min, and incubated with Click-iT^®^ Plus OPP reaction cocktail for copper (I)-catalyzed azide-alkyne cycloaddition with an azide-linked Fluor-488 group.

The third method was to use AHA, an azide analog of methionine, to metabolically label newly synthesized proteins. The assay was performed according to Click-iT^®^ AHA Alexa Fluor^®^ 488 Protein Synthesis Assay Kit (Life Technologies). Before treatments, cells were cultured in a medium without methionine (Life Technologies) for 30 min to deplete endogenous methionine. SN3-L6 and AHA (50 μM) together were added to the cells for 2 h. Then cells were fixed in 3.7% formaldehyde, permeabilized in 0.5% Triton^®^ X-100, and incubated with the reaction cocktail for azide-alkyne cycloaddition with an alkyne Fluor-488 group.

For both the OPP and AHA labeling methods, images were taken using a ZEISS LSM700 confocal microscope with a 63× objective.

### Cell Transfection

For Foxp1, Foxp4, Hsf1, or Erf knockdown, plasmids including scr-RNA, Foxp1-shRNA, Foxp4-shRNA, Hsf1-shRNA, or Erf-shRNA were designed and transfected into Neuro-2a cells, respectively, together with a GFP plasmid in a ratio of 2:1 using Lipofectamine LTX (Life Technologies). The sequences of shRNAs used in this study were as follows:

Foxp1-shRNA, GACAGTGGATGAAGTAGAGTT;
Foxp4-shRNA, CAGGAGAAGTAATGACAAATT;
Hsf1-shRNA, GCTCATTCAGTTCCTGATC;
Erf-shRNA, CACCGGCTTGTTCTTCATCAT;
Scr-RNA, CAACAAGATGAAGAGCACCAA.

### Statistical Analysis

All experiments were performed independently at least three times. For quantification of Western blot result, the densitometric measurement of each band (or whole bands for puromycin blots) was performed using Quantity One 1-D Analysis Software (Bio-Rad). Neurite number and length were quantified by ImageJ. Fluorescent intensity was measured by Image-Pro Plus 6. Statistically significant differences between two groups and multiple groups were determined by Student’s *t*-test and the analysis of variance (ANOVA) followed by Bonferroni’s Multiple Comparison Test, respectively. Data were expressed as mean ± SEM (standard error of the mean). The levels of significance were set at *P* < 0.05.

## Results

### SN3-L6 Potently Promotes Neuronal Differentiation and Neurite Outgrowth

Our previous findings showed that the ability of SN3-L6 in promoting neuronal differentiation is similar to retinoic acid (RA), a well-known neuronal morphogen for potent neural fate induction (Tang et al., 2016). Here, we further compared more aspects of differentiation induced by SN3-L6 and RA, and we also included another widely used neuronal differentiation inducing factor dbcAMP for the comparison (**Figure 1B**). Notably, in all aspects we examined, including differentiation rate (**Figure 1C**), neurite number (**Figure 1D**), longest neurite length (**Figure 1E**), and total neurite length (**Figure 1F**), SN3-L6 exhibited equal activities as RA did. By contrast, although dbcAMP also promoted neuronal differentiation to some extent, its effects were much weaker than SN3-L6 and RA. Moreover, SN3-L6 had minimal effects on cell viability or cell growth up to 50 μM (**Figure 1G**).

### SN3-L6 Upregulates Proteins in Transcriptional and Translational Regulation, and Protein Metabolism

**FIGURE 2:**
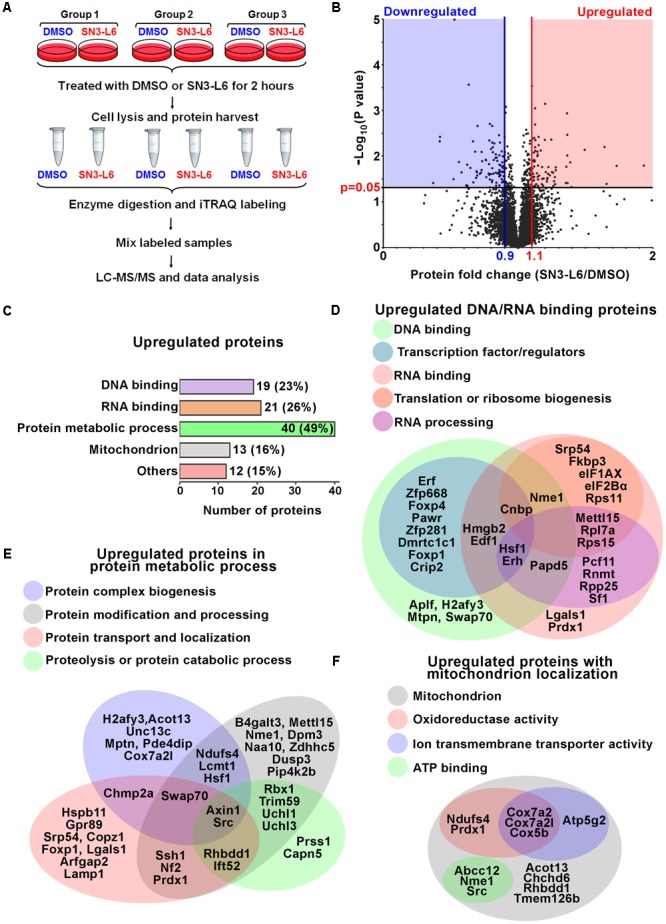
SN3-L6 upregulates proteins in DNA and RNA binding, protein metabolism and mitochondrial localization. **(A)** A schematic of the experimental procedures for isobaric tag for relative and absolute quantitation (iTRAQ) analysis. **(B)** A Volcano plot is drawn to show the distribution of all proteins identified by LC-MS/MS. Upregulated and downregulated proteins are defined by 1.1- and 0.9-fold change, respectively, with *P*-value smaller than 0.05 (distributed in red and blue areas of the plot, respectively). **(C)** Numbers of proteins that are involved in DNA binding, RNA binding, protein metabolic process and mitochondrion localization. Venn diagrams are drawn to show classifications of individual proteins involved in DNA and RNA binding **(D)**, protein metabolic process **(E)**, and mitochondrion localization **(F)**.

We have shown that SN3-L6 is capable of inducing signaling changes at very early stages, i.e., ERK (extracellular-regulated kinase), Akt and calcium-dependent signaling activation was prominently evident at 15 min of treatment and lasted for up to 120 min (Tang et al., 2016). This suggests that cellular reprogramming by SN3-L6 at early stages may be the key for differentiation induction. To this end, we took advantage of the iTRAQ method to investigate whether a pattern of proteomic changes occur in cells to respond to SN3-L6 during the first two hours of treatment (**Figure 2A**).

More than 6500 proteins were identified by the Liquid Chromatography (LC) – Electrospray Ionization (ESI) Tandem MS (MS/MS) method following sample labeling (**Supplementary Table S1**). As expected, robust protein level changes (more than 50% increase or decrease) were rarely observed during such a short time window of treatment (**Figure 2B**). By setting 10% change as a threshold, 81 and 86 proteins were identified as upregulated and downregulated proteins, respectively (**Supplementary Table S2**). We used Gene Ontology tools from PANTHER (Protein Analysis Through Evolutionary Relationships) together with Functional Annotation Tool from DAVID Bioinformatics Resources to analyze the classification of these proteins. Intriguingly, 85% of the upregulated proteins (69 out of 81) belonged to the following categories, DNA and RNA binding, protein metabolic processes and mitochondrial localization (**Figure 2C**). In the DNA binding category, 13 upregulated proteins are transcription factors/regulators, suggesting a reprogram of the transcriptional regulation network (**Figure 2D**). Importantly, seven of the upregulated proteins have been shown to promote neuronal differentiation or fate determination of NSPCs. For example, the Foxp of proneural transcription factors Foxp1 and Foxp4 have been extensively studied for neuronal fate determination *in vivo* and *in vitro* (Konstantoulas et al., 2010; Rousso et al., 2012; Adams et al., 2015; Precious et al., 2016). Other upregulated transcription regulators known for neuronal differentiation include Cnbp (Weiner et al., 2011), Erf (Janesick et al., 2013), Hsf1 (Takaki et al., 2006; Uchida et al., 2011), doublesex and mab-3 related (Dmrt) like protein Dmrtc1c1 (De Clercq et al., 2016), and high mobility group box 2 (Hmgb2) (Bagherpoor et al., 2017; Bronstein et al., 2017). Moreover, nucleoside diphosphate kinase A (Nme1), a nuclease which shows transcriptional regulation activities, is also involved in neuron fate specification (Owlanj et al., 2012). Therefore, it appears that SN3-L6 reprograms the transcription regulation network of Neuro-2a cells in favor of the induction of neuronal differentiation.

For the RNA binding proteins (21 proteins), we did further subcategorization and found that most of them are involved in translation (10 proteins) and RNA processing (10 proteins; **Figure 2D**). These include translation initiation factor (eIF) 1AX and eIF2Bα, 40 and 60S ribosome proteins (Rps11, Rps15, and Rpl7a), and mRNA processing factors such as splicing (splicing factor; Sf1), capping (mRNA cap guanine-N7 methyltransferase; Rnmt1), and polyadenylation (Non-canonical polyadenylation RNA polymerase Papd5 and Cleavage and polyadenylation factor subunit homolog Pcf11) factors (**Figure 2D**). Therefore, it seems that SN3-L6 activates a subgroup of proteins for mRNA maturation, ribosome biogenesis and translational regulation. We interestingly found that this notion may be supported by the subcategorization result of protein metabolic processes, in which four subgroups are found, protein biogenesis, protein modification, protein transport and localization, and proteolysis (**Figure 2E**). This means that the whole protein turnover processes, which occur in sequence following translation, are all positively regulated by SN3-L6. In accordance with the elevated translation and protein turnover, which requires energy, a portion of mitochondrial proteins that have oxidoreductase activity, ion transmembrane transporter activity and ATP binding property were also upregulated (**Figure 2F**), presumably to facilitate producing energy needed for protein synthesis. Collectively, the proteomic analysis of SN3-L6 treated cells revealed an intriguing reprogramming of transcription regulators, together with elevated mRNA translation, protein synthesis and metabolism.

### SN3-L6 Promotes Global mRNA Translation

**FIGURE 3:**
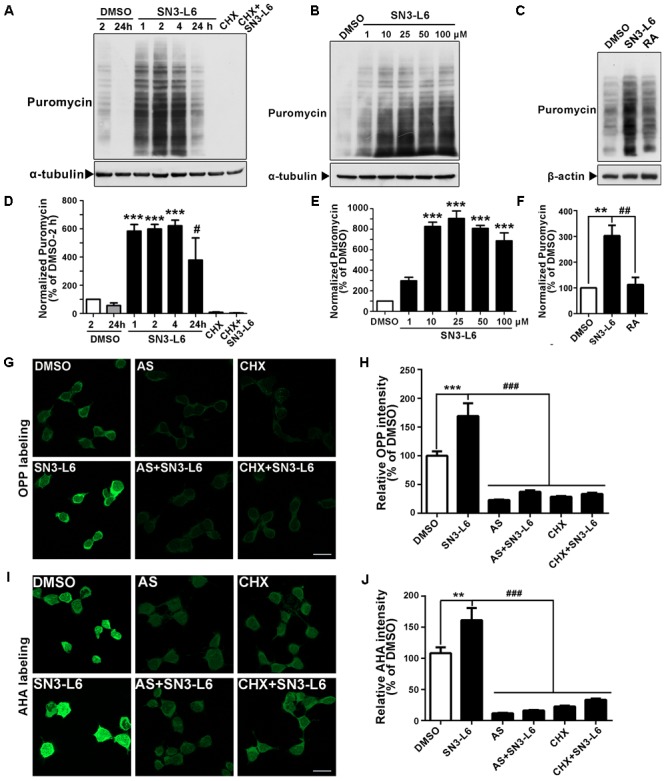
SN3-L6 promotes mRNA translation. Representative Western blots showing the effects on puromycin incorporation by SN3-L6 (25 μM) at different treatment time **(A)** and different concentrations (1–100 μM; **B**). α-tubulin was used as a loading control. Cycloheximide (CHX) (12 μM) was used to block translation. **(C)** Comparison of the effects on puromycin incorporation by SN3-L6 (25 μM) and RA (10 μM). β-actin was used as a loading control. **(D)** Quantification of puromycin incorporation levels of **(A)**. ^∗∗∗^*P* < 0.001, SN3-L6-2 h vs. DMSO-2 h; ^#^*P* < 0.05, SN3-L6-24 h vs. DMSO-24 h. **(E)** Quantification of puromycin incorporation levels of **(B)**. ^∗∗∗^*P* < 0.001, SN3-L6 vs. DMSO. **(F)** Quantification of puromycin incorporation levels of **(C)**. ^∗∗^*P* < 0.01, SN3-L6 vs. DMSO; ^##^*P* < 0.01, RA vs. SN3-L6. **(G)** Representative images of O-propargyl-puromycin (OPP) labeling of newly synthesized polypeptides treated by SN3-L6 (25 μM). **(H)** Quantification of OPP signal intensity. **(I)** Representative images of L-azidohomoalanine (AHA) labeling of newly synthesized proteins. **(J)** Quantification of AHA signal intensity. For **(H,J)**, ^∗∗^*P* < 0.01, ^∗∗∗^*P* < 0.001, SN3-L6 vs. DMSO; ^###^*P* < 0.001, anisomycin (AS) or CHX single or co-treatment with SN3-L6 vs. SN3-L6. All data shown in this figure are presented as mean ± SEM from at least three independent experiments. Statistical analysis was subjected to one-way ANOVA with Bonferroni multiple comparison test. Scale bars, 20 μm.

To verify whether SN3-L6 indeed promotes protein synthesis, we first took advantage of puromycin labeling, a well-used non-radioactive method to monitor global mRNA translation (Schmidt et al., 2009). Puromycin is an aminonucleoside antibiotic structurally similar to aminoacyl-tRNAs, thus can be incorporated into the C terminus of nascent polypeptide chains. Intriguingly, SN3-L6 induced rapid and robust puromycin labeling (**Figure 3A**). This induction remained obvious at 24 h of treatment, although the extent of puromycin labeling was weaker comparing to 1–4 h treatment (**Figure 3A**). The puromycin signals were remarkably blocked after the addition of CHX, an inhibitor that stops peptide elongation of translation (**Figures 3A,D**), suggesting that SN3-L6-induced puromycin labeling was indeed come from the upregulation of translation. We then tested the effects of different concentrations (1–100 μM) of SN3-L6 on inducing protein synthesis. It appeared that 10 μM showed similar activity as 25 μM did, and any concentration above that did not further promote puromycin labeling (**Figures 3B,E**). Importantly, this translational activation activity of SN3-L6 was rather unique, as RA had no effect on puromycin labeling (**Figures 3C,F**), although the two compounds promoted neuronal differentiation similarly.

To visualize protein synthesis in individual cells, we used OPP, an alkyne analog of puromycin which is incorporated into newly synthesized polypeptides as puromycin. Alexa Fluor^®^ 488 picolyl azide was then added to ligate with OPP through picolyl azide-alkyne reaction (known as the click reaction), so that newly synthesized proteins could be detected *in situ* by imaging. We found that 2 h treatment of SN3-L6 obviously induced more protein synthesis indicated by the much brighter signal of OPP (**Figures 3G,H**). To test translation in a more physiological context, we used AHA labeling method. AHA, an analog of methionine with an azido moiety, is incorporated into newly synthesized proteins in methionine-free culture condition. Alkyne-modified Alexa Fluor^®^ 488 was then ligated to the AHA-containing proteins. Similarly, the AHA method also showed that SN3-L6 robustly increased protein synthesis (**Figures 3I,J**). The translation inhibitors AS and CHX substantially blocked OPP and AHA signals (**Figures 3G–J**). These three methods collectively show that SN3-L6 is a new compound that has robust translational activation activity.

### Translation Activated by SN3-L6 Requires MEK and mTOR Activity and Is Cap-Dependent

**FIGURE 4:**
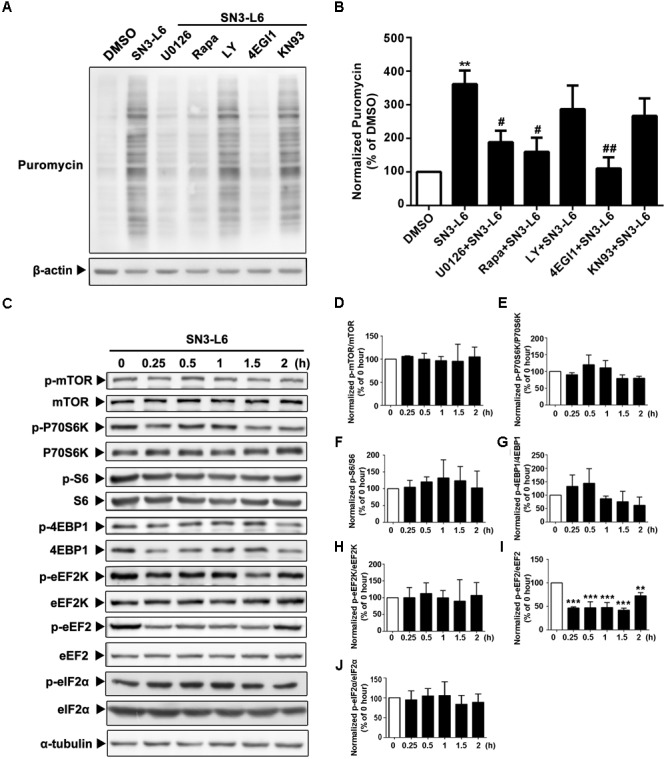
Translation activated by SN3-L6 requires MEK and mTOR activity and is cap-dependent. **(A)** Neuro-2a cells were treated with SN3-L6 (25 μM) together with MEK inhibitor (U0126; 10 μM), mTORC1 inhibitor [Rapamycin (Rapa); 10 μM], PI3K inhibitor (LY294002; LY; 10 μM), cap-dependent translation initiation inhibitor (4EGI1; 50 μM) or CaMKII inhibitor (KN93; 0.5 μM) for 2 h. Nascent polypeptides were examined by puromycin incorporation. **(B)** Quantification of puromycin incorporation levels. ^∗∗^*P* < 0.01, SN3-L6 vs. DMSO; ^#^*P* < 0.05, ^##^*P* < 0.01, co-treatment vs. SN3-L6. **(C)** SN3-L6 was added in Neuro-2a cells for indicated time points (0–120 min). Cells lysates were subjected to Western blot analysis for the phosphorylated- and total-forms of different signaling molecules. **(D–J)** Quantification of the phosphorylation levels of different molecules. ^∗∗^*P* < 0.01, ^∗∗∗^*P* < 0.001, SN3-L6 treatment vs. no treatment (0 h). All data shown in this figure are presented as mean ± SEM from at least three independent experiments. Statistical analysis was subjected to one-way ANOVA with Bonferroni multiple comparison test.

The most common way of eukaryotic mRNA translation is to initiate translation from the 7-methyl-G(5′)ppp(5′)N-modification at the 5′-UTR of mRNA, known as the cap structure (Hinnebusch et al., 2016). mTORC1 (mTOR complex 1)-mediated pathway is the major signaling pathway known to activate cap-dependent translation. We previously showed that SN3-L6 activates Akt and ERK (Tang et al., 2016), both of which act as the upstream molecules that lead to mTORC1 activation. Interestingly, mTORC1 inhibitor Rapamycin and ERK inhibitor U0126 completely blocked SN3-L6 effect on translation, whereas phosphatidylinositide 3-kinase (AKT kinase) inhibitor LY294002 had no effect (**Figures 4A,B**). Consistently, pharmacological disruption of the initiation protein complex at the cap structure by 4EGI1 inhibited SN3-L6 effect (**Figures 4A,B**). Blockade of CaMKII (Ca^2+^/calmodulin-dependent protein kinase-II), a kinase activated by SN3-L6 but is not directly related to translation, had no effect on translation induced by SN3-L6 (**Figures 4A,B**). These results suggest that SN3-L6 effect may be through mTORC1-mediated cap-dependent translation.

We then performed a detailed investigation on major signaling molecules downstream of mTORC1 that are important for translation. Unexpectedly, neither mTORC1-S6K (ribosomal protein S6 kinase)-S6 axis, which leads to ribosome biogenesis, nor mTORC1-4EBP1 axis, which leads to cap-dependent assembly of translation initiation protein complex, was activated by SN3-L6 (**Figures 4C–G**). For mTORC1-eEF2K (eukaryotic elongation factor 2 kinase)-eEF2 axis, which regulates the nascent peptide elongation, only eEF2 was activated, indicated by the decrease of phosphorylation at Thr^56^ (**Figures 4C,I**). Interestingly, although eEF2K is by far the only known kinase that phosphorylates Thr^56^ of eEF2, the phosphorylation of eEF2K itself did not change (**Figures 4C,H**). Moreover, eIF2α, which is important for translation initiation and is activated independent of mTORC1, was not regulated by SN3-L6 (**Figure 4J**). Together, among the mTORC1-mediated signaling pathways we examined so far, eEF2 appears to be the only downstream molecule that is activated by SN3-L6.

### Inhibition of Translation Blocks Neuronal Differentiation Induced by SN3-L6

**FIGURE 5:**
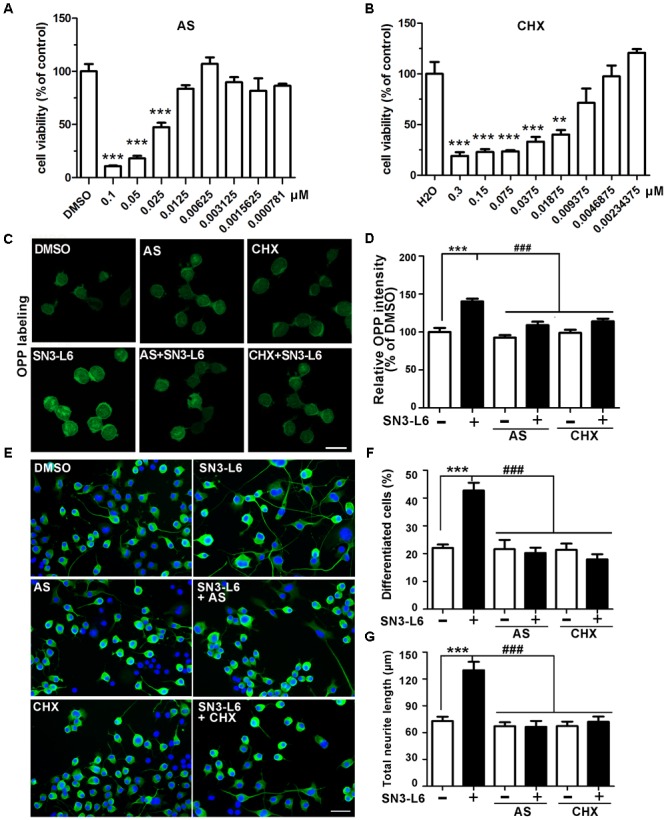
Neuronal differentiation induced by SN3-L6 depends on translation. Neuro-2a cells were treated with different concentrations of AS **(A)** and CHX **(B)** for 48 h. Cell viability was performed by MTT assay. ^∗∗∗^*P* < 0.001, ^∗∗^*P* < 0.01, AS vs. DMSO or CHX vs. H_2_O. **(C)** Representative images of OPP labeling of newly synthesized polypeptides treated with SN3-L6 together with AS (12.5 nM) or CHX (9 nM). Scale bar, 20 μm. **(D)** Quantification of OPP signal intensity. ^∗∗∗^*P* < 0.001, SN3-L6 vs. DMSO; ^###^*P* < 0.001, AS or CHX vs. SN3-L6. **(E)** Representative images of Neuro-2a cells treated with SN3-L6 (25 μM), AS (12.5 nM) or CHX (9 nM). Neurites and nuclei were visualized using β-tubulin III antibody (green) and DAPI (blue), respectively. Scale bar, 50 μm. Percentage of differentiated cells **(F)** and total neurite length **(G)** are quantified. ^∗∗∗^*P* < 0.001, SN3-L6 vs. DMSO; ^###^*P* < 0.001, AS or CHX single or co-treatment with SN3-L6 vs. SN3-L6. All data shown in this figure are presented as mean ± SEM from at least three independent experiments. Statistical analysis was subjected to one-way ANOVA with Bonferroni multiple comparison test.

We have shown that SN3-L6 promotes a quick and robust induction of global translation. The immediate question would be whether translation was the essential mechanism required for SN3-L6 to induce neuronal differentiation. We addressed this question by addition of the translation inhibitors AS and CHX. As chronic inhibition of translation could be harmful for cells, we tried a series concentrations of AS and CHX for two-day treatment in the differentiation medium. Cell viability assay showed that much lowered doses of these two inhibitors were appropriate for studying neuronal differentiation (up to 12.5 nM AS and 9.3 nM CHX vs. 0.5 μM AS and 12 μM CHX in acute treatments; **Figures 5A,B**). We then verified that these doses (12.5 nM AS and 9 nM CHX) hardly diminished basal translation, but effectively blocked SN3-L6-dependent translation (**Figures 5C,D**). Indeed, SN3-L6 was not able to induce more cells to differentiation, nor did it promote neurite extension in the presence of either inhibitor (**Figures 5E–G**). Thus, SN3-L6-induced neuronal differentiation is dependent on its translational activation activity.

### SN3-L6 Translationally Upregulates Foxp1, Foxp4, Erf, and Hsf1

**FIGURE 6:**
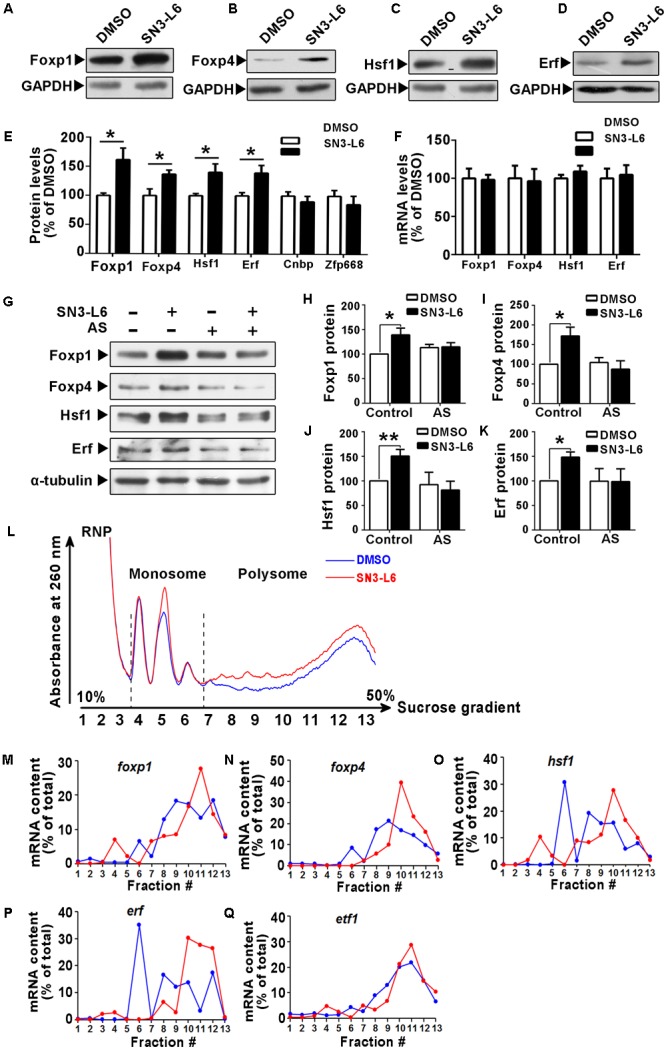
SN3-L6 translationally upregulates Foxp1, Foxp4, Hsf1, and Erf. Western blot analysis shows that SN3-L6 (25 μM) increased protein expressions of transcription factors Foxp1 **(A)**, Foxp4 **(B)**, Hsf1 **(C)**, and Erf **(D)**. GAPDH was used as a loading control. **(E)** Statistical analysis of the protein level changes of Foxp1, Foxp4, Hsf1, and Erf. Error bars represent SEM from three independent experiments, unpaired Student’s *t*-test; ^∗^*P* < 0.05. **(F)** SN3-L6 did not affect mRNA transcript levels of Foxp1, Foxp4, Hsf1, and Erf as revealed by representative RT-qPCR results. Data are normalized by GAPDH mRNA levels and are presented as mean ± SEM from four independent experiments, unpaired Student’s *t*-test. **(G)** Western blot analysis showing that the translation inhibitor AS (0.5 μM) blocked SN3-L6-induced upregulation of Foxp1, Foxp4, Hsf1, and Erf. α-tubulin was used as a loading control. **(H–K)** Quantification of protein levels. Data are presented as mean ± SEM from four independent experiments. ^∗^*P* < 0.05, ^∗∗^*P* < 0.01, SN3-L6 vs. DMSO, unpaired Student’s *t*-test in group. **(L)** Polysome profiles from Neuro-2a cells show that SN3-L6 treatment for 2 h increased global translation. RT-qPCR analysis showing amounts of *foxp1* **(M)**, *foxp4* **(N)**, *hsf1* **(O)**, *erf* **(P)**, and *etf1* (gene of eRF; **Q**) mRNAs in different fractions. RNP, ribonucleoprotein.

The next question is what proteins are synthesized in response to SN3-L6. Among the upregulated proteins shown from the iTRAQ result, we were particularly interested in the transcription factors, as reprogram of transcription network is the key to induce neuronal differentiation. Therefore, we selected the top five increased transcription factors for validation, which are Cnbp, Erf, Zfp668, Foxp4, and Hsf1. Foxp1 (13% increase) was also included, as it belongs to the same family of Foxp4. Indeed, Foxp1, Foxp4, Hsf1, and Erf all showed significantly increased protein levels upon 2 h treatment of SN3-L6 (**Figures 6A–E**). However, we failed to detect changes of Cnbp and Zfp688 proteins (**Figure 6E**). The increase of Foxp1, Foxp4, Hsf1, and Erf occurred at protein levels, as none of them showed changes at mRNA levels (**Figure 6F**). Consistently, application of AS canceled the upregulation of these transcription factors by SN3-L6 (**Figures 6G–K**), suggesting that the increase of their protein levels is translation-dependent. To further confirm that SN3-L6 induces translation of mRNAs of these transcription factors, we employed polysome profiling combined with RT-qPCR analysis of individual mRNAs. Consistent with the elevation of global mRNA translation by SN3-L6, we observed a remarkable increase of polysome-associated mRNAs, indicating more active general translation upon SN3-L6 treatment (**Figure 6L**). By examining different fractions of ribonucleoprotein (containing non ribosome-bound mRNAs), monosome and polysome, we found that mRNAs of Foxp1, Foxp4, Hsf1, and Erf all showed a shift of distribution to heavier polysome fractions (**Figures 6M–P**), which indicates that these mRNAs are more translational active in SN3-L6-treated cells. As a control, mRNA of eukaryotic translation termination factor 1 (eRF1), which terminates translation, did not show obvious change of mRNA distribution (**Figure 6Q**). These results confirmed that Foxp1, Foxp4, Hsf1, and Erf are translationally upregulated by SN3-L6.

### Knockdown of Foxp1, Foxp4, Hsf1, or Erf Inhibits SN3-L6-Induced Neuronal Differentiation

**FIGURE 7:**
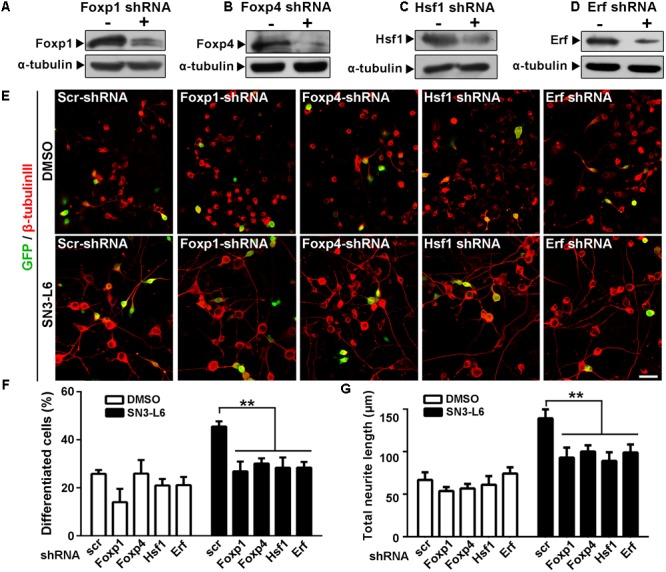
Knockdown of Fox1, Foxp4, Hsf1, or Erf inhibits SN3-L6 effects on neuronal differentiation. **(A–D)** Neuro-2a Cells were transfected with scramble (scr) shRNA or shRNA of Foxp1, Foxp4, Hsf1, or Erf for 24 h. Western blot analysis shows that the knockdown of the individual transcription factors was successful. α-tubulin was used as a loading control. **(E)** Cells were co-transfected with GFP and scr-shRNA of individual shRNAs of Foxp1, Foxp4, Hsf1, or Erf. 24 h later, SN3-L6 was added to the cells to allow differentiation for 48 h. Neurites was visualized using β-tubulin III antibody (red). Transfected cells were indicated by GFP signals, Scale bar, 50 μm. Differentiation rate **(F)** and total neurite length **(G)** were measured. ^∗∗^*P* < 0.01, different shRNAs vs. scr-shRNA, mean ± SEM from three independent experiments. Statistical analysis was subjected to one-way ANOVA with Bonferroni multiple comparison test.

To test whether protein synthesis of Foxp1, Foxp4, Hsf1, and Erf are essential for SN3-L6 induced neuronal differentiation, we constructed individual shRNAs of these transcription factors to interfere their mRNAs, and the protein knockdown efficiency were confirmed (**Figures 7A–D**). Indeed, knockdown of each transcription factor all substantially reduced the effect of SN3-L6 on differentiation rate and neurite growth (**Figures 7E–G**). Thus, translation-dependent expression of Foxp1, Fop4, Hsf1, and Erf is essentially required for SN3-L6-induced neuronal differentiation.

### SN3-L6 Promotes Neuronal Differentiation in Neural Progenitor Cells

**FIGURE 8:**
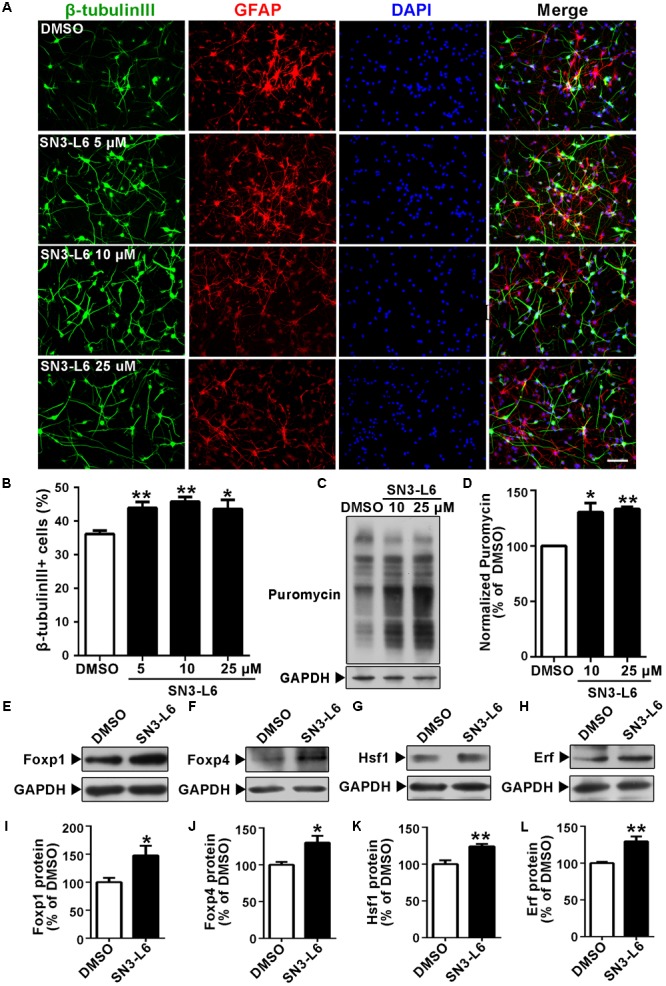
SN3-L6 promotes neuronal differentiation in cortical neural progenitor cells (NPCs). **(A)** Primary NPCs were differentiated for 5 days in the presence of DMSO or different concentrations of SN3-L6. Neurons and glial cells were immunostained with β-tubulin III (green) and GFAP (red), respectively. Nuclei were counterstained with DAPI. Scale bar, 50 μm. **(B)** Quantification of β-tubulin III^+^ cells differentiated from NPCs. Data are presented as mean ± SEM from four independent experiments. ^∗^*P* < 0.05, ^∗∗^*P* < 0.01, SN3-L6 vs. DMSO, one-way ANOVA with Bonferroni multiple comparison test. **(C)** Effects of SN3-L6 (10 or 25 μM) on puromycin incorporation. **(D)** Puromycin levels were quantified and presented as percentage of change comparing to DMSO. ^∗^*P* < 0.05, ^∗∗^*P* < 0.01, SN3-L6 vs. DMSO. Error bars represent SEM from three independent experiments, one-way ANOVA with Bonferroni multiple comparison test. **(E–H)** Western blot analysis of protein levels of Foxp1, Foxp4, Hsf1, and Erf in NPCs after treatment of SN3-L6 (10 μM). **(I–L)** Quantification of protein level changes comparing to DMSO. Error bars represent SEM. Three independent experiments were performed. ^∗^*P* < 0.05, ^∗∗^*P* < 0.01, SN3-L6 vs. DMSO, unpaired Student’s *t*-test.

To reveal whether SN3-L6 has abilities to promote neurogenesis in NSPCs, we examined the compound in primary cortical NPC cultures. Indeed, SN3-L6 treatment for 5 days enhanced neural induction, indicated by higher percentages of cells positive for the neuronal marker β-tubulin III (**Figures 8A,B**). For the three concentrations we tested (5, 10, and 25 μM), the induction of differentiation was similar (∼45% in SN3-L6 group vs. ∼37% in control group). Consistent with the observations in Neuro-2a cells, SN3-L6 promoted global translation indicated by the more incorporation of puromycin, although the increase degree of puromycin levels was much less than in Neuro-2a cells (∼40% increase in NPCs vs. several folds increase in Neuro-2a cells; **Figures 8C,D**). One possible reason for this discrepancy could be that the translational machinery in NPCs is already in a more active state that is not as easily further activated as in Neuro-2a cells. We then examined whether the four transcription factors identified in Neuro-2a cells are also upregulated by SN3-L6 in NPCs. Indeed, protein levels of Foxp1, Foxp4, Hsf1, and Erf were all increased in NPCs upon 2 h treatment of SN3-L6 (**Figures 8E–L**). Together, these findings provide compelling evidence that SN3-L6, through a novel translation-dependent mechanism that lead to synthesis of a subset of proneural transcription factors, is a new small molecule to induce neuronal differentiation.

## Discussion

It is well established that transcriptional reprogramming leads to neuronal fate determination and differentiation of NSPCs. However, how regulation at translational levels participates in these processes has only started to be understood. Our study reinforces the notion that translational regulation is indeed an essential switch for neuronal differentiation by demonstrating that a small molecule, through translational upregulation of particular transcriptional factors, induces neuronal differentiation. To our best knowledge, this is the first report of a small-molecule compound which promotes neuronal differentiation by targeting translational regulation. We believe that this opens a new avenue for the design of neural induction drugs for stem cell therapy and neural regeneration.

Because of the huge complexity of translational regulation in NSPCs, strategies targeting translation has not been widely considered for engineering cell fate determination and neuronal differentiation. There was one report using a reversible photoregulation approach to switch on or off the translation of H-Ras mRNA, which successfully controls neuronal differentiation (Ogasawara, 2014). Moreover, in muscle stem cells, switching on the global translation by pharmacological activation of eIF2α has been found to trigger muscle cell differentiation (Zismanov et al., 2016). However, chemical manipulations of global translation have not been tried in NSPCs. Our study not only strengthens the theory that mRNA translation controls neuronal differentiation, but also provides the first evidence that small molecule-based stimulation of translational regulation is a powerful approach for engineering neural induction. SN3-L6 quickly activates global mRNA translation in Neuro-2a and NPCs. As a result, a group of neurogenic transcription factors including Foxp1, Foxp4, Hsf1, and Erf are synthesized, driving neuronal fate determination and differentiation. Importantly, Foxp1 and Foxp4 are expressed in developing forebrain and spinal cord, and regulate glutamatergic projection neuron and motor neuron differentiation (Takahashi et al., 2008; Hisaoka et al., 2010; Rousso et al., 2012; Precious et al., 2016). Ectopic expression of Foxp1 in NSPCs has been used to induce motor neuron or dopaminergic neuron identities (Konstantoulas et al., 2010; Adams et al., 2015). In addition, Hsf1 is essential for the adult neurogenesis of hippocampal neurons and olfactory neurons, and Erf is a responsive gene in RA-induced neuronal differentiation. These provide clues to understand the neuronal identity induced by SN3-L6, but a detailed analysis will be required to fully uncover whether SN3-L6-induced differentiation generates mature, functional neurons of specific subtype. To obtain more detailed information of the gene-specific translatome regulated by SN3-L6, sequencing of polysome-associated mRNAs or BONCAT (Bioorthogonal Non-canonical Amino Acid Tagging)-based purification of newly synthesized proteins for proteomic analysis would be required.

Modulation of translation has the advantage to offer a more direct and efficient approach to control protein levels without modifications of the host genome. However, despite there are many translation inhibitors (Garreau de Loubresse et al., 2014), not as many translation activators have been reported. Most of the translation activating compounds identified so far belong to a group of small molecules that target the inhibition of PERK (protein kinase R-like endoplasmic reticulum kinase)-elF2α pathway (Hetz et al., 2013; Smith and Mallucci, 2016; Halliday et al., 2017), which switches on translation initiation. Another major group is the inhibitors of eEF2K and its upstream kinases such as AMP-activated protein kinase (AMPK); blocking either kinase leads to dephosphorylation-dependent activation of eEF2 and potentiates translation elongation (Ma et al., 2014). Both groups of the translation activating compounds have been studied in postmitotic neurons, but none have been tested in NSPCs for neuronal differentiation activities. However, these compounds should be used with cautions: PERK is important for the unfolded protein response which controls cell adaptation and proteostas is in different physiological and pathological contexts, so its inhibition may diminish the cell adaptation capacity; eEF2K inhibitors do not always lead to lowered eEF2 phosphorylation and other possible targets may be involved (Chen et al., 2011; Bender et al., 2016). The present study provides a new compound that shows robust translational activation activity, which may act through ERK-mTORCI-eEF2 pathway. This adds another tool for studying translational regulation in different biological processes. A crucial future direction is to uncover the detailed molecular actions of SN3-L6. Chemical modifications of SN3-L6 with engineered fluorescent or biotin group would be extremely helpful for revealing the cellular localization and molecular target(s) of the compound.

## Author Contributions

YL, XZ, XH, YP, XM, and LS performed the experiments. Z-XH, YW, W-MC, and W-CY provided the compound SN3-L6 and analyzing methods. FL contributed to obtaining the polysome profiling experiments. JX contributed to data interpretation and figure organization. YL, XZ, W-CY, W-MC, and LS analyzed the data. YL, XZ, and LS wrote the manuscript.

## Conflict of Interest Statement

The authors declare that the research was conducted in the absence of any commercial or financial relationships that could be construed as a potential conflict of interest.

## Footnotes

1 http://pantherdb.org/

2 https://david.ncifcrf.gov/

## Supplementary Material

TABLE S1 Total proteins identified by iTRAQ.

TABLE S2 Up- and down-regulated proteins.

## Abbreviations

AHA: L-azidohomoalanine
AS: anisomycin
CHX: cycloheximide
Cnbp: CCHC-Type zinc finger nucleic acid binding protein
DAPI: 49,6-diamidino-2-phenylindole
dbcAMP: dibutyl cyclic AMP
eEF: eukaryotic translation elongation factor
eIF: eukaryotic translation initiation factor
Erf: ETS2 repressor factor
Foxp: forxhead box protein P family
GO: Gene Ontology
Hsf1: heat shock transcription factor 1
iTRAQ: isobaric tag for relative and absolute quantitation
NPCs: cortical neural progenitor cells
NSPCs: neural stem/progenitor cells
OPP: O-propargyl-puromycin
RA: retinoic acid
